# Short term association between air pollution (PM_10_, NO_2_ and O_3_) and secondary spontaneous pneumothorax

**DOI:** 10.1038/s41598-020-68831-4

**Published:** 2020-07-16

**Authors:** Tania Marx, Nadine Bernard, Anne-Laure Parmentier, Marc Puyraveau, Berenger Martin, Madeleine Gantelet, Jean-Baptiste Pretalli, Jean-Charles Dalphin, Frédéric Mauny, Thibaut Desmettre

**Affiliations:** 1 Emergency Department, CHU Besançon, 3 boulevard Alexandre Fleming, 25030 Besançon, France; 2 Laboratory Chrono-Environnement, UMR 6249 Centre National de la Recherche Scientifique, 16 route de Gray, 25000 Besançon, France; 3 Laboratory TheMA, UMR 6049 Centre National de la Recherche Scientifique, 16 route de Gray, 25000 Besançon, France; 4 uMETh-Inserm Centre Investigation Clinique 1431, CHU Besançon, 2 place Saint-Jacques, 25030 Besançon, France; 5 Respiratory Medicine Department, CHU Besançon, 3 boulevard Alexandre Fleming, 25030 Besançon, France; 6 Université Bourgogne Franche-Comté, 32 avenue de l’Observatoire, 25000 Besançon, France

**Keywords:** Diseases, Respiratory tract diseases, Environmental impact, Health care, Epidemiology

## Abstract

Secondary spontaneous pneumothorax (SSP) occurs in the context of underlying pulmonary disease. Our objectives were to estimate the relationship between SSP and short term air pollution exposure with nitrogen dioxide (NO_2_), ozone (O_3_) and particulate matter with a diameter ≤ 10 μm (PM_10_). Patients with SSP were included between June 1, 2009 and May 31, 2013, in 14 Emergency Departments in France. In this case–crossover design study, PM_10,_ NO_2_, and O_3_ data were collected hourly from monitoring stations. Quantitative values, fast increase in air pollutant concentration, and air quality threshold exceedance were retained. These assessments were calculated for each of the 4 days prior to the event (Lag 1–Lag 4) for all case and control period, and for the entire exposure period. A total of 135 patients with SSP were included, with a mean age of 55.56 (SD 18.54) years. For short term exposure of PM_10_, NO_2_ and O_3_, no differences were observed between case and control periods in terms of quantitative values of air pollutant exposure (P > 0.68), fast increase in concentration (P > 0.12) or air quality threshold exceedance (P > 0.68). An association between O_3_ exposures cannot be ruled out, especially when considering the Lag 2 prior to the event and in warm seasons.

## Introduction

The link between air pollution and respiratory disease has been established, even if the role of each pollutant has not yet been clearly identified. Li et al.^1^ demonstrated, with a case–crossover design, that air pollutants have a short term effect on a variety of acute respiratory diseases. Particulate matter with diameter ≤ 10 μm and ≤ 2.5 µm (PM_10_ and PM_2.5_), nitrogen dioxide (NO_2_), sulfur dioxide (SO_2_) and carbon monoxide (CO) had positive associations with outpatient visits for upper respiratory tract infection, acute bronchitis, community acquired pneumonia, acute exacerbation of bronchiectasis and acute exacerbation of chronic obstructive pulmonary disease (COPD). Two meta-analyses confirmed these results for COPD related emergency department visits, hospital admission and mortality^2,3^. Concerning ozone (O_3_), a positive association with outpatient visits for acute exacerbation of asthma and COPD was demonstrated^1,4,5^. For other respiratory diseases, such as pneumothorax, very few studies have documented the relationship with air pollution. Relationships were reported between the occurrence of primary spontaneous pneumothorax (PSP) and interactions with higher values of air pollutants and other meteorological parameters, such as atmospheric pressure, temperature and humidity^6–9^_._ Abul et al.^10^ showed a link between an increase in O_3_ and the occurrence of PSP. In a previous study, we demonstrated a lack of connection between PSP and NO_2_ and PM_10_ exposure, but could not rule out an association between O_3_ exposure and PSP^11^. Secondary spontaneous pneumothorax (SSP) occurs in the context of underlying pulmonary disease. Among several recognized causes of SSP, the most common aetiology is COPD, which accounts for 70–80%^12^. Therefore, it is important to analyse a potential link between SSP and air pollution. The objectives of this study were: (1) to estimate the relationships between SSP and short term air pollution exposure with PM_10_, NO_2_ and O_3_; and (2) to investigate a time lag effect between exposure and the occurrence of SPP.

## Methods

**Figure 1:**
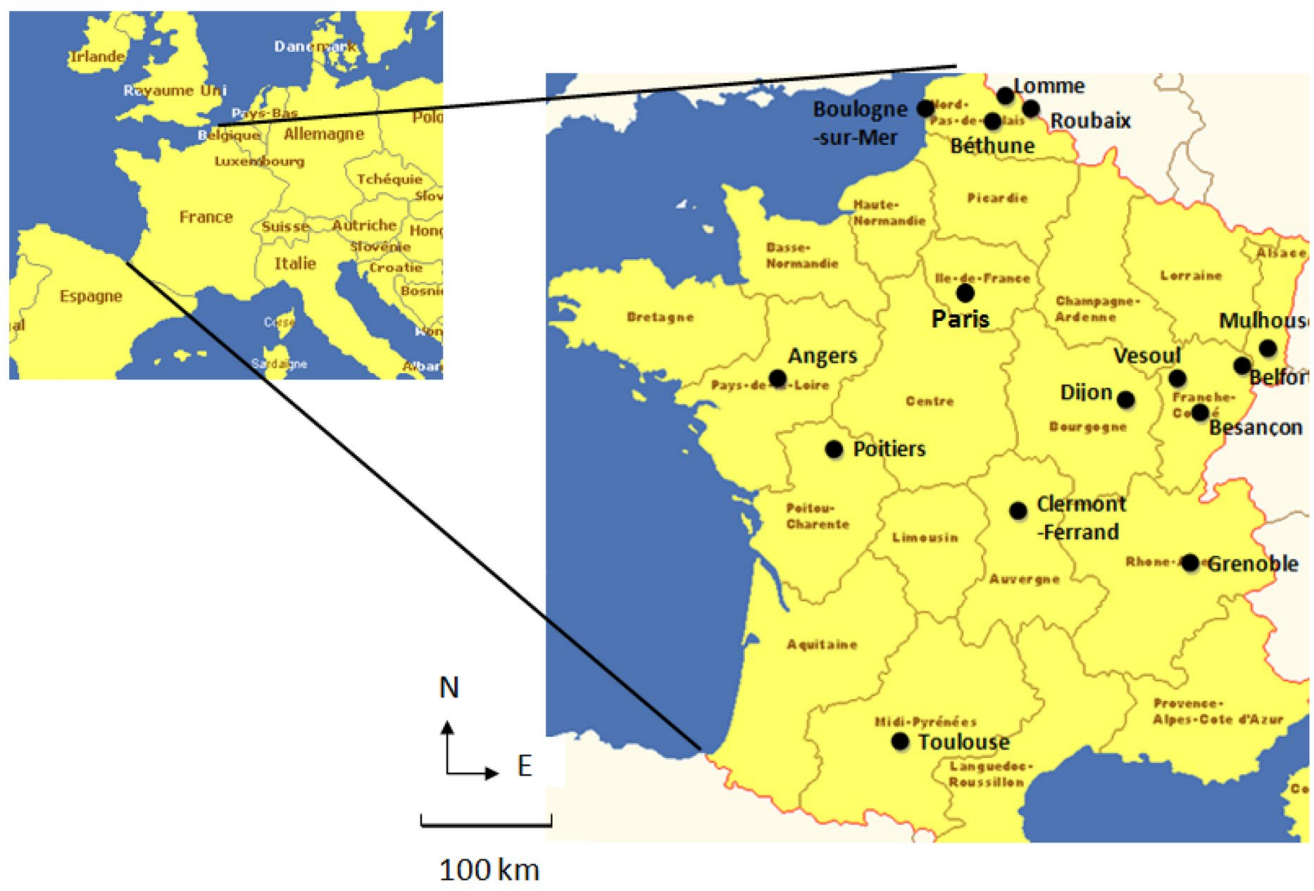
Geographical location of the 14 participating centers. Seven university teaching hospitals, namely Angers, Besançon, Clermont-Ferrand, Dijon, Grenoble, Poitiers and Toulouse; and seven general, non-academic hospitals, namely Belfort, Béthune, Boulogne sur Mer, Lomme, Roubaix, Mulhouse and Vesoul. Maximal distance between two hospitals: north–south 798 km; east–west 593 km (maps created with the software Microsoft Word 2013 15.0 and Paint 6.1 from Microsoft Windows 7 Professionnel https://www.microsoft.com/fr).

The design of this study is a time-stratified case–crossover^13^. This design is used to study environmental conditions, particularly air pollution^14–17^. This study implicated Emergency Departments from 14 medium-sized cities distributed over all the territory of France (Fig. 1).

For each subject, a case period was compared to multiple other control periods dispersed over the month concerned by the occurrence of the event. The date of admission to the Emergency Department determined the date of occurrence of the SSP. Case period was defined as the period leading up to the date of admission, concerning the total time during which the subject was exposed to the risk of occurrence of SPP. A period of 4 days was retained, i.e., Lag 1–Lag 4, before the occurrence of the event. Control periods were defined as the same weekdays as the case period within the same month. The exposure period corresponded as all case and control periods for each subject.

### Study population

All patients with pneumothorax aged over 18 years and who provided written informed consent from, the period of June 1, 2009, to May 31, 2013, were included. Patients with traumatic and PSP were excluded. Recurrent episodes, defined as a second episode of SSP during the period of inclusion, were excluded. Subjects living more than 30 km (19 miles) from an air pollutant monitoring station, or in a different study area were also excluded.

Data collected from the Emergency Department medical records were the following: age, sex, date of admission, home address, smoking status, and history of COPD, emphysema, pneumothorax and asthma. All research was performed in accordance with relevant regulations and informed consent was obtained from all the participants. The study was approved by the French National authority for the protection of privacy and personal data (CNIL) on December 23, 2014 under the number 913594.

### Environmental data

Air pollution exposure was assessed using measurements provided by the French National AASQA Network, which is accredited by the French Ministry for the Environment and whose quality is guaranteed by ISO/TC146 standard for air quality. Measurements, data control and the maintenance of the instrumentation are standardized while respecting French and European standards. Hourly mean concentration of NO_2_, O_3_ and PM_10_ were collected from each monitoring station of the study area. The number of stations ranged between one to nine per area (mean 5.9). Each patient’s home address was used to estimate the exposure to air pollutants for the case and control periods. The association between the patient’s home address and the appropriate monitoring station was determined by the local AASQA team. To select the most representative station for measuring the exposure of each patient, different parameters were considered: the distance between the geolocated address and the station, the geomorphological situation and the proximity of significant sources of air pollution.

Three exposure assessments were selected. First, the maximum and mean absolute quantitative values of PM_10_, NO_2_ and O_3_ were determined. Second, the fast increase in air pollutant concentration was calculated, which was defined as a positive difference in concentration occurring in an interval of 3 h, noted as ∆_3 h._ For each hour, ∆_3 h_ was calculated as the difference between the concentrations measured at hour h, minus the concentration measured at hour h-3. A fast increase was retained if a ∆_3 h_ exceeded: 20 µg/m^3^ for PM_10_, or 40 µg/m^3^ for NO_2_ and O_3_. This threshold value was chosen as the third quartile of the observed distribution of the ∆_3 h._

Finally, the air quality threshold exceedance was defined by the European legislation on air quality^18^. The legal thresholds for each air pollutant were: PM_10_ daily average = 50 µg/m^3^, NO_2_ hourly average = 200 µg/m^3^, O_3_ 8-h average = 120 µg/m^3^.

To highlight a lag effect, these assessments were calculated for each of the 4 days before the occurrence of SSP for all the case and control periods, and for the entire exposure period (Lag 1–Lag 4).

### Statistical analysis

A conditional logistic regression was used to analyse the relationship between the exposure to air pollution for each case and control period. To control the false discovery rate with a high number of exposure indices, a correction for multiple testing was realized with Benjamini Yekutieli’s adjustement, which is less conservative than a Bonferroni correction^19–21^. To verify that the effects of concentrations of pollutants were not modified by individual factors, sensitivity analyses were performed with age, sex, smoking status, history of pulmonary disease (COPD, emphysema, pneumothorax, and asthma) and centre. Complementary analyses were also performed with warm seasons (spring and summer) versus cool seasons (autumn and winter) to consider the variation of O_3_ concentration with the mount of sunlight.

Statistical analyses were performed using SAS, version 9.4 (SAS Institute Inc., Cary, NC, USA). A P value < 0.05 was considered statistically significant.

## Results

### Participants

**Figure 2:**
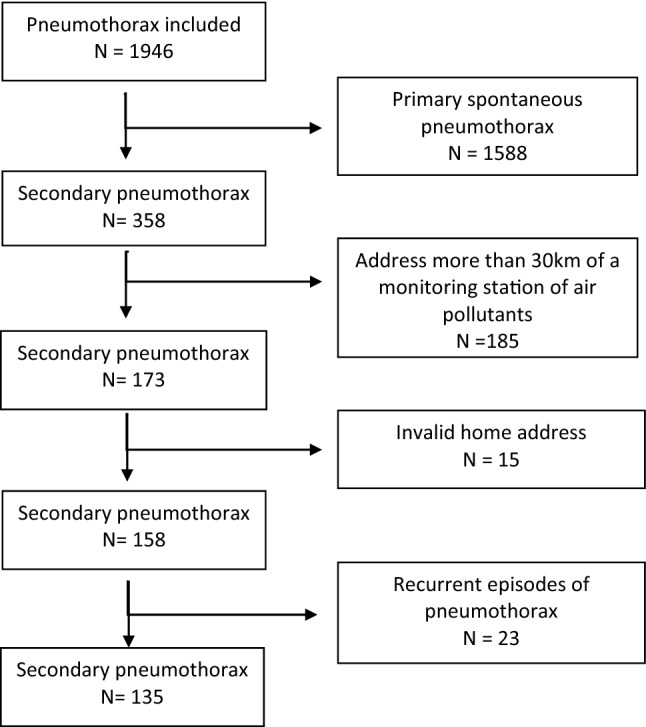
Flow chart.

From June 1, 2009, to May 31, 2013, were admitted in the 14 Emergency department 1946 patients for pneumothorax. Among them, 358 patients presented a SSP. After excluding subjects living more than 30 km (19 miles) from an air pollutant monitoring station or with a recurrent episodes, 135 SSP were included (Fig. 2). The mean age of the population was 55.56 years (standard derivation 18.54 years). The male/female ratio was 4.4. Approximately 60% of the population presented with COPD or emphysema, and 39% declared a previous onset of pneumothorax. Current smokers accounted for 54%. Half of the SSPs were admitted in spring and summer seasons (Table 1).

**Table 1:** Baseline characteristics of study population.

N = 135	Mean (SD) or N (%)
**Age** (years)	55.56 (18.54)
**Sex**	
Male	110 (81.48)
Female	25 (18.52)
**History of COPD**	82 (60.74)
**History of emphysema**	78 (57.78)
**History of pneumothorax**	53 (39.26)
**History of asthma**	8 (5.93)
**Smoking status**^a^	
Current	73 (54.07)
Never	5 (3.70)
Former	34 (25.19)
**Season**	
Spring	40 (29.63)
Summer	29 (21.48)
Autumn	33 (24.44)
Winter	33 (24.44)

*COPD* chronic obstructive pulmonary disease.

^a^Missing values N = 23.

### Air pollution exposure

The pollutant exposures are presented according to the case and the control status in SI Table A (mean (SD), median, maximum, minimum and quartile) and SI Table B (correlation coefficients between the air pollutants).

The odds ratios associated with PM_10,_ NO_2_ and O_3_ values of maximum and mean concentrations, for each Lag and the entire period exposure are presented in Table 2. The odds ratios associated with at least one fast increase (∆_3 h_) are presented in Table 3. The odds ratios associated with at least one exceedance of air quality threshold are presented in Table 4.

**Table 2:** Association between the maximum and mean concentration pollutants over the entire exposure period, and the occurrence of secondary pneumothorax.

Variables	PM_10_			NO_2_			O_3_		
	OR (95% CI)*	*P* value†	*P*_*cor*_ value‡	OR (95% CI)	*P *value	*P*_*cor*_ value	OR (95% CI)	*P *value	*P*_*cor*_ value
**Maximum**									
Lag 1	0.94 (0.83–1.06)	0.33	1.00	0.95 (0.86–1.05)	0.30	0.95	0.99 (0.89–1.09)	0.80	0.80
Lag 2	0.97 (0.86–1.08)	0.54	1.00	1.02 (0.92–1.14)	0.65	0.95	1.02 (0.93–1.13)	0.67	0.72
Lag 3	0.99 (0.91–1.08)	0.90	1.00	1.04 (0.95–1.14)	0.39	0.95	1.08 (0.98–1.20)	0.12	0.68
Lag 4	0.98 (0.87–1.09)	0.66	1.00	1.02 (0.93–1.13)	0.66	0.95	0.97 (0.88–1.08)	0.60	0.72
Entire period¥	0.96 (0.88–1.05)	0.37	1.00	1.04 (0.94–1.14)	0.50	0.95	1.04 (0.92–1.16)	0.55	0.72
**Mean**									
Lag 1	0.94 (0.78–1.15)	0.56	1.00	0.87 (0.70–1.09)	0.24	0.95	1.02 (0.90–1.15)	0.79	0.79
Lag 2	0.97 (0.80–1.18)	0.77	1.00	1.02 (0.83–1.25)	0.85	0.95	0.99 (0.87–1.12)	0.83	0.83
Lag 3	0.93 (0.77–1.13)	0.46	1.00	0.97 (0.79–1.18)	0.72	0.95	1.03 (0.91–1.16)	0.67	0.72
Lag 4	0.97 (0.82–1.15)	0.71	1.00	0.99 (0.82–1.21)	0.95	0.95	0.95 (0.84–1.07)	0.41	0.68
Entire period	0.91 (0.72–1.15)	0.41	1.00	0.95 (0.72–1.25)	0.71	0.95	0.99 (0.84–1.17)	0.94	0.94

The OR were computed for a 10 µg/m^3^ increase.

*CI* confidence interval.

*Confidence interval without correction for multiple comparison procedure.

^†^For conditional logistic regression.

^‡^Corrected *P* value with Benjamini Yekutieli’s method.

^¥^Entire exposure period = Lag 1 to date Lag 4.

**Table 3:** Association between the exposure to at least one episode of fast increase in air pollutant concentration and the occurrence of secondary pneumothorax.

Variables	OR (95% CI)*	*P* value†	*P*_*cor*_ value‡
**PM**_**10**_** (concentration of 20 µg/m**^**3**^**)**			
Lag 1	1.02 (0.56–1.85)	0.95	1.00
Lag 2	0.96 (0.53–1.73)	0.88	1.00
Lag 3	0.52 (0.27–1.00)	0.05	0.97
Lag 4	1.01 (0.53–1.91)	0.97	1.00
Entire exposure period^¥^	0.84 (0.55–1.29)	0.43	1.00
**NO**_**2**_** (concentration of 40 µg/m**^**3**^**)**			
Lag 1	0.37 (0.18–0.77)	0.01	0.12
Lag 2	1.07 (0.57–1.99)	0.83	0.95
Lag 3	1.22 (0.65–2.29)	0.53	0.95
Lag 4	1.24 (0.65–2.38)	0.52	0.95
Entire exposure period	0.97 (0.59–1.57)	0.89	0.95
**O**_**3**_** (concentration of 40 µg/m**^**3**^**)**			
Lag 1	1.40 (0.84–2.32)	0.20	0.68
Lag 2	1.41 (0.84–2.35)	0.19	0.68
Lag 3	1.26 (0.76–2.10)	0.37	0.68
Lag 4	1.03 (0.61–1.74)	0.91	0.91
Entire exposure period	1.06 (0.69–1.62)	0.79	0.79

*CI* confidence interval.

*Confidence interval without correction for multiple comparison procedure.

^†^For conditional logistic regression.

^‡^Corrected *P* value with Benjamini Yekutieli’s method.

^¥^Entire exposure period = Lag 1 to date Lag 4.

**Table 4:** Association between exposure to at least one air quality threshold exceedance of pollution and the occurrence of secondary pneumothorax.

Variables	OR (95% CI)*	*P* value†	*P*_*cor*_ value‡
**PM**_**10**_** (daily average 50 µg/m**^**3**^**)**			
Lag 1	0.41 (0.11–1.49)	0.17	1.00
Lag 2	0.54 (0.15–1.90)	0.34	1.00
Lag 3	1.00 (0.35–2.88)	1.00	1.00
Lag 4	0.96 (0.34–2.73)	0.94	1.00
Entire exposure period¥	0.82 (0.38–1.78)	0.62	1.00
**O**_**3**_** (8-h average 120 µg/m**^**3**^**)**			
Lag 1	1.69 (0.71–4.02)	0.23	0.68
Lag 2	1.77 (0.63–4.95)	0.28	0.68
Lag 3	1.72 (0.58–5.08)	0.33	0.68
Lag 4	0.91 (0.29–2.81)	0.87	0.87
Entire exposure period	1.36 (0.66–2.79)	0.40	0.68

*CI* confidence interval.

*Confidence interval without correction for multiple comparison procedure.

^†^For conditional logistic regression.

^‡^Corrected *P* value with Benjamini Yekutieli’s method.

^¥^Entire exposure period = Lag 1 to date Lag 4.

Concerning the PM_10_ exposure assessment, the odds ratios associated with values of maximum and mean concentrations across Lag 1–Lag 4 ranged between 0.93 (0.77–1.13) and 0.99 (0.91–1.08) (Table 2). When considering each Lag, the odds ratios associated with PM_10_ values of at least one fast increase (∆_3 h_) ranged between 0.52 (0.27–1.00) and 1.02 (0.56–1.85) (Table 3) ; the odds ratios associated with at least one exceedance of air quality threshold ranged between 0.41 (0.11–1.49) and 1.00 (0.35–2.88) (Table 4).

Concerning the NO_2_ exposure assessment, the odds ratios associated with values of maximum and mean concentrations across Lag 1–Lag 4 ranged between 0.87 (0.70–1.09) and 0.99 (0.82–1.21) (Table 2). When considering each Lag, the odds ratios associated with NO_2_ values of at least one fast increase (∆_3 h_) ranged between 0.37 (0.18–0.77) and 1.24 (0.65–2.38) (Table 3). No exceedance of air quality threshold of NO_2_ was observed.

Concerning the O_3_ exposure assessment, the odds ratios associated with values of maximum and mean concentrations across Lag 1–Lag 4 ranged between 0.95 (0.84–1.07) and 1.02 (0.93–1.13) (Table 2). When considering each Lag, the odds ratios associated with O_3_ values of at least one fast increase (∆_3 h_) ranged between 1.03 (0.61–1.74) and 1.40 (0.84–2.32) (Table 3) ; the odds ratios associated with at least one exceedance of air quality threshold ranged between 0.91 (0.29–2.81) and 1.77 (0.63–4.95) (Table 4).

The complementary analyses O_3_ values separately performed on warn and cold seasons are presented in SI Figure A and SI Table C.

No significant association between the occurrence of SSP and the air pollutant exposure assessment were observed. All P_Corr_ values were superior to 0.12.

## Discussion

In this study, no significant association between the occurrence of SSP and short term exposure to PM_10,_ NO_2_, and O_3_ were identified for quantitative absolute values, fast increase in air pollutants and peak of pollution. Nevertheless, a link between O_3_ and the occurrence of the event cannot be ruled out.

Our study had some strengths. We were careful to avoid selection bias. During the study period, all patients with a diagnosis of pneumothorax [International Classification of Diseases (ICD) 10th revision code = J93] were retrospectively identified by medical informatics queries of the emergency unit databases in the participating centres. The diagnosis of SSP (ICD code J93.12) was checked by research assistants in the medical files of the Emergency Department’s admissions database.

Concerning the air pollution exposure for each patient, a partnership was realized with the French National AASQA Network. The aim was to identify the appropriate monitoring station in relation to each patient’s address. Considering the geomorphological situation and the sources of primary pollutant, allows us to determine the most reliable level of air pollutant exposure.

The time-stratified case–crossover design, which is an advanced matching technique, makes it possible to take into account the characteristics of individual patients. This design is recognized to consider temporal trends and to reduce differential errors due to the measure of exposure. This methodology is specifically adapted to the analysis of associations between rapid onset disease with a short induction period, and acute exposure^14,22^.

To the best of our knowledge, our study is the first in the literature to evaluate the link between SSP and air pollution. Patients with SSP have mainly underlying chronic lung diseases, such as COPD. In their study on the epidemiology of pneumothorax, Gupta et al.^12^ demonstrated that COPD causes 70–80% of SSP cases. In our study, approximately 60% of patients with SSP presented an underlying COPD or an emphysema. A significant impact between acute exacerbation of COPD and air pollution was demonstrated^1–3,5^. Long-term exposure to air pollutants is significantly associated with increasing emphysema^23,24^. With these chronic inflammatory diseases, these population are most vulnerable and present a greater risk of pulmonary complications. A diffuse histopathological change in the lung parenchyma under the visceral pleural could lead to the occurrence of a pneumothorax. Diffuse increased porosity at the periphery of both lungs has been identified, which could be associated with localized rupture of a bleb, or a bulla^25,26^. Short-term O_3_ exposure could induce airway hyperresponsiveness, increase oxidant stress and stimulate inflammatory responses in the respiratory system associated with a chronic inflammatory disease, which could lend credence to the hypothesis of a stronger link between SSP and air pollutants^27^.

Nevertheless, in our study, a link between O_3_ exposure and SSP cannot be ruled out. In the literature, Park et al.^7^ showed that increased concentrations of O_3_ on two previous days before the occurrence of PSP are significantly associated. Huang et al.^5^ demonstrated that the strongest association between O_3_ exposure and COPD mortality was found in the moving average concentration from the day of the occurrence of COPD to Lag 2. Yang et al.^28^ concluded at an association among respiratory mortality and increased average O_3_ 2 days before the occurrence of the event. Although non significant, our study results associated with the fast increase of 40 µg/m^3^ and the peak of 8-h average of 120 µg/m^3^ are compatible with the occurrence of SSP and a fast increase O_3_ concentration to Lag 2 prior to the event.

The concentrations of O_3_ are generally higher in warm seasons than in cool seasons. In fact, the concentration and distribution of O_3_ are linked with regard to the main determinants: emissions and sunlight. Primary pollutant emissions from urban traffic include nitrogen oxides, carbon monoxide, and volatile organic compound produce O_3_ through photochemical transformations. A strong correlation between O_3_ concentrations and temperature is established^29^; increasing temperature also increases the emission of volatile organic compound by processes such as evaporation^30^. Ozone concentration increases almost linearly with the ambient temperatures and with greater intensity of sunlight, which is why O_3_ concentrations are higher in warm seasons and in urban areas^29,31,32^. In the literature, Abul et al.^10^ showed an association between the occurrence of PSP and higher levels of O_3_ in the spring. Gryparis et al.^33^ evaluated the relationship between acute effects of O_3_ and respiratory mortality in 23 Europeans cities. O_3_ concentrations are higher during the summer and are associated with an increase respiratory mortality during warmer seasons. Zanobetti et al. and Peng et al.^34,35^ demonstrated the same results for American and Canadian cities. Although our study results are non significant, the ORs concerning the associations between maximum and mean O_3_ concentrations and the occurrence of SSP are higher in warm seasons than in cool seasons for each Lag and for the entire period of exposure before the event. A link between short term O_3_ exposure and these diseases cannot be ruled out in warm seasons.

In this regard, two points merit discussion, namely the seasonality and the associations of different meteorological factors.

In the literature, the occurrence of pneumothorax and respiratory disease seems to have a seasonal effect. However, the results are contradictory. In fact, Park et al. and Aissa et al.^7,36^ demonstrated a greater occurence of pneumothorax in warm versus cold seasons. Schieman et al. and Motono et al.^37,38^ concluded that there was an absence of a significant link between pneumothorax and seasonality. Concerning respiratory diseases, such as COPD, a relationship is established with a higher concentration of O_3_ during the summer^33–35^. However, Chen et al., Yin et al. and Wong et al.^39–41^ showed in East Asia and southern China that O_3_, despite a higher concentration in warm versus cool seasons, had stronger effects on respiratory mortality in cool seasons The main explanation for this finding is the people’s behavior in relation to the climate. American, Canadian and European cities are exposed to a continental climate with extremely cold winters and hot summers with a great range of temperatures. People also spend longer amounts of time outdoors during warm seasons than in cool seasons. They are exposed in warm seasons to higher outdoors O_3_ pollution and pollen concentration, which can increase respiratory disease exacerbation^33^. Southeast Asia has a subtropical climate with mild winter and hot and damp summers. During cool seasons, people are more likely to go outdoors and are also more exposed to O_3_. During warm stay at home with the air conditioner and are also less exposed to O_3_^5,41^. Therefore, the exposition of air pollutants depends on lifestyle and people’s behavior. Climate, with the associations of different meteorological factors, is therefore an interesting parameter to evaluate the occurrence of pneumothorax.

Concerning the different meteorological factors, the associations of air pollutants and different parameters, such as atmospheric pressure, temperature and humidity, were not included in our study. In fact, Bertolaccini et al.^8^ concluded that the occurrence of PSP was significantly triggered by higher daily mean NO_2_, lower daily mean O_3_ and daily decrement of standard variation of temperature and wind speed. In the second study of Bertolaccini et al.^9^, PSP was significantly more likely to occur on warm windy days with high atmospheric pressure and high mean NO_2_ concentrations. Park et al.^7^ demonstrated that increased concentrations of air pollutants associated with an abrupt change in atmospheric pressure were significantly associated with an increased incidence rate of PSP. Temperature and wind speed might affect the transport of air pollutants and allergens, leading to bronchiolar spasm, mucous retention and coughing^7,37^. The association with different factors could have a synergistic effect, and a resulting impact on air pollution. All environmental factors should be taken into account to evaluate their relationship with pneumothorax.

## Conclusions

Our study failed to identify any significant relationship between SSP and short-term PM_10_ and NO_2_ exposure. A link with O_3_ exposures cannot be ruled out, especially when considering 2 days prior to the event and in warm seasons. Therefore, a potentiating effect of different meteorological factors remains to be demonstrated.

## Supplementary information

Supplementary file1 (PDF 357 kb)

## References

[1] Li R, Jiang N, Liu Q, Huang J, Guo X, Liu F (2017). Impact of air pollutants on outpatient visits for acute respiratory outcomes. Int. J. Environ. Res. Public Health.

[2] Bloemsma LD, Hoek G, Smit LAM (2016). Panel studies of air pollution in patients with COPD: Systematic review and meta-analysis. Environ. Res..

[3] DeVries R, Kriebel D, Sama S (2017). Outdoor air pollution and COPD-related emergency department visits, hospital admissions, and mortality: A meta-analysis. COPD.

[4] Zu K, Liu X, Shi L, Tao G, Loftus CT, Lange S (2017). Concentration-response of short-term ozone exposure and hospital admissions for asthma in Texas. Environ. Int..

[5] Huang J, Li G, Xu G, Qian X, Zhao Y, Pan X (2018). The burden of ozone pollution on years of life lost from chronic obstructive pulmonary disease in a city of Yangtze River Delta, China. Environ. Pollut..

[6] Han C, Lim Y-H, Jung K, Hong Y-C (2019). Association between ambient air pollution exposure and spontaneous pneumothorax occurrence. Epidemiol. Camb. Mass..

[7] Park JH, Lee SH, Yun SJ, Ryu S, Choi SW, Kim HJ (2018). Air pollutants and atmospheric pressure increased risk of ED visit for spontaneous pneumothorax. Am. J. Emerg. Med..

[8] Bertolaccini L, Alemanno L, Rocco G, Cassardo C (2011). Air pollution, weather variations and primary spontaneous pneumothorax. J. Thorac. Dis..

[9] Bertolaccini L, Viti A, Boschetto L, Pasini A, Attanasio A, Terzi A (2015). Analysis of spontaneous pneumothorax in the city of Cuneo: Environmental correlations with meteorological and air pollutant variables. Surg Today..

[10] Abul Y, Karakurt S, Bostanci K, Yuksel M, Eryuksel E, Evman S (2011). Spontaneous pneumothorax and ozone levels: Is there a relation?. Multidiscip. Respir. Med..

[11] Marx T, Bernard N, Parmentier A-L, Puyraveau M, Martin B, Gantelet M (2019). Does air pollution really impact the onset of spontaneous pneumothorax? A French case–crossover study. Environ. Int..

[12] Gupta D, Hansell A, Nichols T, Duong T, Ayres JG, Strachan D (2000). Epidemiology of pneumothorax in England. Thorax.

[13] Maclure M (1991). The case–crossover design: A method for studying transient effects on the risk of acute events. Am. J. Epidemiol..

[14] Bateson TF, Schwartz J (1999). Control for seasonal variation and time trend in case–crossover studies of acute effects of environmental exposures. Epidemiology..

[15] Lumley T, Levy D (2000). Bias in the case–crossover design: Implications for studies of air pollution. Environmetrics.

[16] Levy D, Lumley T, Sheppard L, Kaufman J, Checkoway H (2001). Referent selection in case–crossover analyses of acute health effects of air pollution. Epidemiology.

[17] Janes H, Sheppard L, Lumley T (2005). Overlap bias in the case–crossover design, with application to air pollution exposures. Stat. Med..

[18] Ministère de l’écologie, de l’énergie, du développement durable et de la mer, en charge des technologies vertes et des négociations sur le climat. Code de l’environnement. Décret n°2010–1250 relatif à la qualité de l’air. Section 1 : Surveillance de la qualité de l’air ambiant Article R221–1. 2010.

[19] Benjamini Y, Hochberg Y (1995). Controlling the false discovery rate: A practical and powerful approach to multiple testing. J. R. Stat. Soc. Ser. B Methodol..

[20] Benjamini Y, Yekutieli D (2001). The control of the false discovery rate in multiple testing under dependency. Ann. Stat..

[21] Reiner A, Yekutieli D, Benjamini Y (2003). Identifying differentially expressed genes using false discovery rate controlling procedures. Bioinformatics.

[22] Janes H, Sheppard L, Lumley T (2005). Case–crossover analyses of air pollution exposure data: Referent selection strategies and their implications for bias. Epidemiology.

[23] Adar SD, Kaufman JD, Diez-Roux AV, Hoffman EA, D’Souza J, Stukovsky KH (2015). Air pollution and percent emphysema identified by computed tomography in the Multi-Ethnic study of Atherosclerosis. Environ. Health Perspect..

[24] Wang M, Aaron CP, Madrigano J, Hoffman EA, Angelini E, Yang J (2019). Association between long-term exposure to ambient air pollution and change in quantitatively assessed emphysema and lung function. JAMA.

[25] Noppen M, Dekeukeleire T, Hanon S, Stratakos G, Amjadi K, Madsen P (2006). Fluorescein-enhanced autofluorescence thoracoscopy in patients with primary spontaneous pneumothorax and normal subjects. Am. J. Respir. Crit. Care Med..

[26] Tschopp J-M, Marquette C-H (2017). Spontaneous pneumothorax: Stop chest tube as first-line therapy. Eur. Respir. J..

[27] Mudway IS, Kelly FJ (2000). Ozone and the lung: A sensitive issue. Mol. Aspects Med..

[28] Yang C, Yang H, Guo S, Wang Z, Xu X, Duan X (2012). Alternative ozone metrics and daily mortality in Suzhou: The China Air Pollution and Health Effects Study (CAPES). Sci. Total Environ..

[29] Bloomer BJ, Stehr JW, Piety CA, Salawitch RJ, Dickerson RR (2009). Observed relationships of ozone air pollution with temperature and emissions. Geophys. Res. Lett..

[30] Stockwell WR, Lawson CV, Saunders E, Goliff WS (2012). A review of tropospheric atmospheric chemistry and gas-phase chemical mechanisms for air quality modeling. Atmosphere.

[31] Baklanov A, Molina LT, Gauss M (2016). Megacities, air quality and climate. Atmos. Environ..

[32] Bernard NL, Gerber MJ, Astre CM, Saintot MJ (1999). Ozone measurement with passive samplers: Validation and use for ozone pollution assessment in Montpellier, France. Environ. Sci. Technol..

[33] Gryparis A, Forsberg B, Katsouyanni K, Analitis A, Touloumi G, Schwartz J (2004). Acute effects of ozone on mortality from the “air pollution and health. Am. J. Respir. Crit. Care Med..

[34] Zanobetti A, Schwartz J (2008). Mortality displacement in the association of ozone with mortality. Am. J. Respir. Crit. Care Med..

[35] Peng RD, Samoli E, Pham L, Dominici F, Touloumi G, Ramsay T (2013). Acute effects of ambient ozone on mortality in Europe and North America: Results from the APHENA study. Air Qual. Atmos. Health..

[36] Aissa S, Maoua M, Selmi S, Benzarti W, Gargouri I, Abdelghani A (2019). Influence of weather conditions on the onset of spontaneous pneumothorax in the region of Sousse (Tunisia): Analysis of time series. BioMed Res. Int..

[37] Schieman C, Graham A, Gelfand G, McFadden SP, Tiruta C, Hill MD (2009). Weather and chinook winds in relation to spontaneous pneumothoraces. Can. J. Surg. J. Can Chir..

[38] Motono N, Maeda S, Honda R, Tanaka M, Machida Y, Usuda K (2018). Atmospheric temperature and pressure influence the onset of spontaneous pneumothorax. Clin. Respir. J..

[39] Chen R, Yin P, Meng X, Liu C, Wang L, Xu X (2017). Fine particulate air pollution and daily mortality. A nationwide analysis in 272 Chinese cities. Am. J. Respir. Crit. Care Med..

[40] Yin P, Chen R, Wang L, Meng X, Liu C, Niu Y (2017). Ambient ozone pollution and daily mortality: A nationwide study in 272 Chinese cities. Environ. Health Perspect..

[41] Wong CM, Ma S, Hedley AJ, Lam TH (2001). Effect of air pollution on daily mortality in Hong Kong. Environ. Health Perspect..

