# Supplementary material for: Short term association between air pollution (PM10, NO2 and O3) and secondary spontaneous pneumothorax
Source: Sci Rep. 2020 Jul 16;10:11823. doi: 10.1038/s41598-020-68831-4 (PMC7366720; doi:10.1038/s41598-020-68831-4)
Supplement: Supplementary file 1 — Supplementary file1 (PDF 357 kb) [file 41598_2020_68831_MOESM1_ESM.pdf]

SUPPLEMENTARY INFORMATION

Title

Short term association between air pollution (PM10, NO2 and O3) and secondary spontaneous pneumothorax.

Authors

Tania Marx, Nadine Bernard, Anne-Laure Parmentier, Marc Puyraveau, Berenger Martin, Madeleine Gantelet, Jean-Baptiste Pretalli, Jean-Charles Dalphin, Frédéric Mauny and Thibaut Desmettre.

Tables

Table A. Distribution of the air pollutants, according to the case and control status, during the exposure period

Variables	Mean (SD)		Median		Q1; Q3		Min; Max	
	Case	Control	Case	Control	Case	Control	Case	Control
PM_{10}								
Lag 1	22.23 (10.79)	23.23 (13.19)	19.88	19.52	14.83 ;28.12	14.66 ; 28.46	1 ;86.8	0 ;140.5
Lag 2	22.1 (11.12)	22.59 (12.45)	20.02	19.47	13.53 ;28.04	14.06 ;27.52	0 ;90	0 ;143
Lag 3	21.82 (11.54)	22.41 (12.20)	19.29	19.23	13.29 ;26.3	14.22 ;28.08	0 ;161	0 ;428
Lag 4	21.72 (12.41)	22.13 (12.81)	18.31	19.40	13.54 ;26.76	13.78 ;26.38	0 ;112	0 ;131
NO_2								
Lag 1	21.53 (12.16)	22.59 (13.81)	19.50	19.79	11.65 ;29.83	11.74 ;30.77	0 ;176	0;148.8
Lag 2	21.73 (13.35)	21.46 (14.27)	19.02	17.87	11.17 ;32.5	10.14 ;29.19	0 ;141.7	0 ;164
Lag 3	21.39 (13.08)	21.71 (13.89)	18.69	18.12	11.54 ;28.58	11.17 ;30.50	0 ;167	0 ;189.8
Lag 4	21.24 (14.35)	21.35 (12.27)	17.26	18.19	10.72 ;28.75	11.03;29.33	0 ;135	0 ;136
O_3								
Lag 1	49.89 (24.67)	49.38 (21.45)	49.60	51.73	30.90 ;66.08	33.94 ;64.77	0 ;161	0 ;168

Lag 2	49.23 (23.34)	49.54 (21.70)	51.08	49.71	33.06 ;64.90	35.59 ;65.20	0 ;168	0 ;168
Lag 3	49.53 (23.24)	48.83 (21.90)	52.31	51.47	33.67 ;66.73	32.42 ;63.04	0 ;175	0 ;165.7
Lag 4	49.78 (22.76)	50.94 (22.78)	50.12	51.76	33.0 ;67.26	34.17 ;65.75	0 ;143	0 ;160

Table B: Correlation coefficients between the air pollutants average concentration, during the exposure period

	NO_2				PM_{10}			
	Lag 1	Lag 2	Lag 3	Lag 4	Lag 1	Lag 2	Lag 3	Lag 4
O_3								
Lag 1	-0.40	-0.38	-0.38	-0.34	-0.12	-0.11	-0.11	-0.07*
Lag 2	-0.38	-0.40	-0.37	-0.31	-0.15	-0.12	-0.09	-0.04*
Lag 3	-0.36	-0.41	-0.41	-0.38	-0.13	-0.14	-0.10	-0.05*
Lag 4	-0.33	-0.34	-0.37	-0.38	-0.08*	-0.09	-0.06*	-0.05*
PM_{10}								
Lag 1	0.55	0.45	0.33	0.25				
Lag 2	0.42	0.58	0.48	0.31				
Lag 3	0.26	0.37	0.55	0.45				
Lag 4	0.21	0.24	0.38	0.54				

*Pearson correlation coefficients, with *P* value > 0.05

Table C : Ozone and the occurrence of secondary pneumothorax during the exposure period for the different seasons.

	Spring and summer seasons			Autumn and winter seasons		
Variables	OR (95% CI)*	*P* value†	P_{cor} value‡	OR (95% CI)	*P* value	P_{cor} value
Maximum						
Lag 1	1.0 (0.89-1.13)	0.99	0.99	0.96 (0.81-1.14)	0.66	0.76
Lag 2	1.06 (0.94-1.20)	0.32	0.32	0.96 (0.82-1.12)	0.59	0.76
Lag 3	1.14 (1.00-1.30)	0.05	0.23	1.01 (0.87-1.17)	0.91	0.91
Lag 4	1.08 (0.94-1.23)	0.27	0.27	0.85 (0.74-0.99)	0.04	0.65
Exposure period	1.09 (0.94-1.25)	0.24	0.24	0.93 (0.76-1.15)	0.52	0.76
Mean						
Lag 1	1.03 (0.87-1.23)	0.72	0.72	1.00 (0.83-1.20)	1.00	1.00
Lag 2	1.09 (0.90-1.31)	0.39	0.39	0.91 (0.76-1.08)	0.27	0.65
Lag 3	1.13 (0.95-1.37)	0.17	0.23	0.94 (0.79-1.11)	0.47	0.76
Lag 4	1.02 (0.86-1.21)	0.79	0.79	0.88 (0.733-1.05)	0.14	0.65
Exposure period	1.13 (0.89-1.43)	0.32	0.32	0.88 (0.70-1.11)	0.29	0.65

Fast increased (concentration of 40µg/m^3)						
Lag 1	1.25 (0.70-2.25)	0.45	0.45	1.96 (0.71-5.39)	0.19	0.65
Lag 2	1.33 (0.72-2.45)	0.37	0.37	1.62 (0.65-4.04)	0.30	0.65
Lag 3	1.29 (0.68-2.44)	0.43	0.43	1.20 (0.51-2.84)	0.67	0.76
Lag 4	1.37 (0.73-2.55)	0.33	0.33	0.54 (0.19-1.52)	0.25	0.65
Exposure period	1.46 (0.83-2.59)	0.19	0.23	0.67 (0.34-1.34)	0.26	0.65
Peak of pollution (8-hour average 120 µg/m^3)						
Lag 1	1.57 (0.63-3.93)	0.33	0.33	3.46 (0.21-55.78)	0.38	0.72
Lag 2	1.97 (0.69-5.64)	0.20	0.23	/	/	/
Lag 3	1.72 (0.58-5.08)	0.33	0.33	/	/	/
Lag 4	0.97 (0.31-3.03)	0.95	0.95	/	/	/
Exposure period¥	1.32 (0.62-2.80)	0.46	0.46	1.81 (0.16-20.01)	0.63	0.76

The OR for maximum and mean concentration were computed for a 10 µg/m^3 increase

Definition of abbreviations: CI = confidence interval

*Confidence interval without correction for multiple comparison procedure.

† For conditional logistic regression.

‡ Corrected *P* Value with Benjamini Yekutieli's method.

¥ Exposure period = Lag 1 to date Lag 4.

Figures

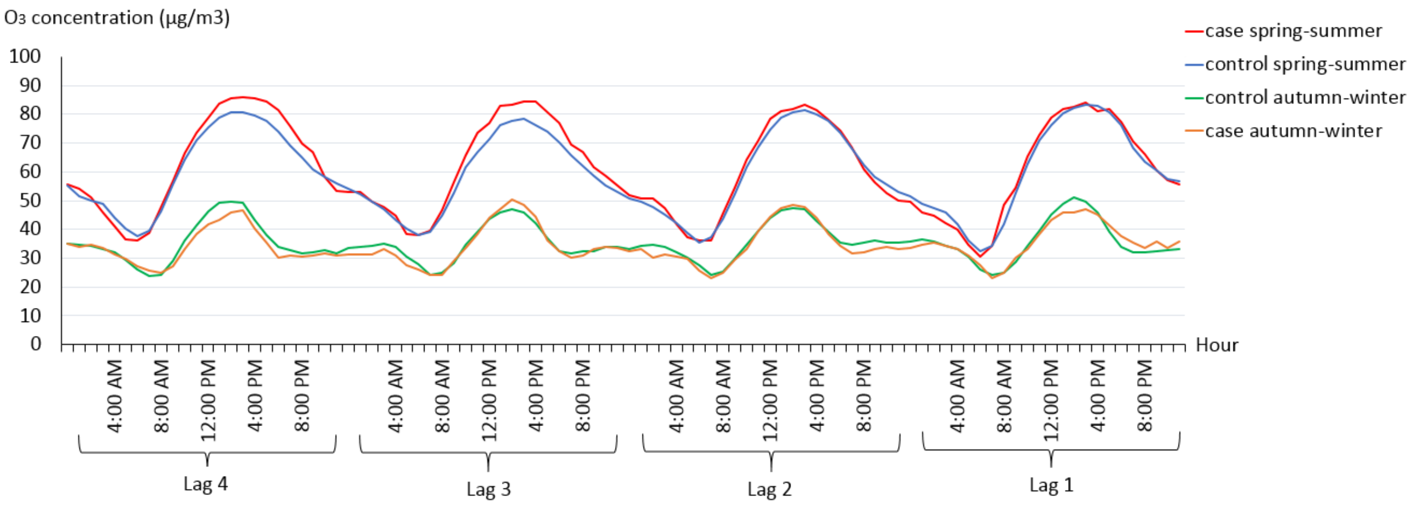

Figure A : Hourly mean concentration of ozone (O_3) according to the four days on the case and controls period, before the occurrence of secondary pneumothorax, for the different seasons.
